# The impact of HIV infection on surgical gastrointestinal diseases at the Princess Marina Hospital, Gaborone, Botswana: a cross-sectional study

**DOI:** 10.11604/pamj.2023.46.72.39140

**Published:** 2023-10-27

**Authors:** Alemayehu Ginbo Bedada

**Affiliations:** 1 Department of Surgery, Faculty of Medicine, University of Botswana, Princess Marina Hospital, Gaborone, Botswana

**Keywords:** CD4 Count, HIV infection, surgical gastrointestinal diseases

## Abstract

**Introduction:**

various gastrointestinal diseases affect surgical patients. Literature on the burden and outcomes of surgical gastrointestinal diseases in a high HIV infection prevalence is scares. This study aimed to investigate this topic at the Princess Marina Hospital.

**Methods:**

medical records of patients admitted with surgical gastrointestinal diseases to adult surgical wards were reviewed from August 2017 to July 2018. Demographics, date of admission and discharge, HIV status, CD4 count, and outcomes were analyzed.

**Results:**

six-hundred and ninety-eight (698) patients with known HIV infection status and surgical gastrointestinal diseases were admitted. HIV+ patients contributed 274 (39.3%). Among HIV+, females contributed 147 (53.6%). Symptomatic gallbladder stone disease was significantly higher in HIV- patients, p=0.008; while anal cancers, p=0.001, anal warts, p=0.001, and perianal infections and fistulae, p=0.010 were significantly higher in HIV+ patients. Overall, surgical site infections were recorded in 15 (2.1%) and mortalities in 43 (6.2%). The mortality rate was higher in HIV+ than in HIV- patients, p=0.048. The total number of surgical procedures and median hospital stays among HIV- and HIV+ patients were not statistically significant, p=0.868 and p=0.249 respectively. The total number of complications, p=0.338, mortality, p=0.149, and median hospital stay, p=0.181, among HIV+ patients based on CD4 count, < 200 vs. > 200, were not significantly different.

**Conclusion:**

symptomatic gallbladder stone diseases were significantly higher in HIV- patients; while anal cancer, anal warts, and perianal infections and perianal fistulae were significantly higher in HIV+ patients. HIV+ patients had a significantly higher mortality rate than HIV- patients, and this needs further investigation.

## Introduction

Human immunodeficiency virus (HIV) infection, prevalent in many sub-Saharan African countries, has a dramatic effect on surgical practice [1-4]. HIV+ patients are subject to diseases that affect immune-competent and immunosuppressed patients. The prevalence of HIV infection in surgical practice is higher than the prevalence in the general population of sub-Saharan countries. This is in part due to the surgically-treatable septic and neoplastic conditions which are common in immunodeficient patients [5]. The prevalence of HIV infection in medical and gynecological wards is higher than the prevalence in surgical wards [6]. Surgical gastrointestinal diseases that affect HIV+ patients include acute abdomen, intestinal obstructions, gastrointestinal perforations, sclerosing cholangitis, acalculous cholecystitis, appendicitis, early age and aggressive colorectal adenocarcinoma, anal ulcers, perianal infections and fistulae, anogenital warts, anal squamous cell carcinoma, unusual perianal opportunistic infections, non-Hodgkin´s lymphoma, Kaposi´s sarcoma, and lymphoma [1,7,8]. Advances in highly active antiretroviral therapy (HAART) reduce viral proliferation and significantly decrease the rate of septic and opportunistic complications in HIV+ patients and improve their survival [1,7,9-13]. The improved survival resulted in a steady increase in the number of HIV infected patients requiring surgical care [1,5]. About 20%-25% of HIV+ patients require operative procedures during their lifetime [14].

Multiple factors determine surgical outcomes in HIV+ patients, including the patient´s age, nutritional status, the degree of immunodeficiency, presence of surgical infection, preoperative white cell count, CD4 count, serum albumin level, degree of emergency, type of anesthesia, type of operation, and operation in a contaminated surgical field [15,16]. Major operations may increase the probability of developing new infections or worsen a preexisting infection by aggravating immunosuppression [15]. The literature provides mixed reports regarding the outcomes of HIV- and HIV+ patients in surgical care. Outcomes range from a higher post-operation sepsis [9,15,17] in HIV+ patients to the absence of significant differences in operative outcomes including duration of wound healing, surgical site infection (SSI), wound dehiscence, number of complications, and length of hospital stays among HIV- and HIV+ patients [10,18]. Weledji *et al*. questioned the effect of HAART on the incidence of anorectal pathologies [1]. Cacala *et al*. reported the absence of significant outcome differences in a heterogeneous group of surgical inpatients concerning their CD4 counts [18]. Although HIV infection is rampant in sub-Saharan African countries, literature is limited in depicting the burden and outcomes of surgical gastrointestinal diseases in high HIV prevalent low- and middle-income countries [2,3]. Botswana with a total population of about 2.3 million has a high HIV infection prevalence of 23.0% [19]. In 2016, Botswana adopted a ‘treat all´ strategy as a national HIV management protocol [20]. This study was designed to investigate the pattern, burden, and outcomes of surgical gastrointestinal diseases admitted to adult surgical wards in a high HIV infection setup at the Princess Marina Hospital (PMH), Gaborone, Botswana: comparing HIV- and HIV+ patients and HIV+ patients based on their CD4 count.

## Methods

**Study design and setting:** this is a retrospective cross-sectional quantitative study that was conducted from August 2017 to July 2018 at the adult surgical wards of the PMH, the largest tertiary public and teaching hospital in Gaborone, Botswana.

**Study population:** all adult surgical patients', aged 13 years old and older (hospital policy), medical records, with the diagnosis of surgical gastrointestinal diseases and known HIV status were included in the study. No patients´ medical record was excluded.

**Data collection:** the data was collected prospectively upon patients' discharge. The medical records were reviewed retrospectively and the data variables were captured in an Excel spreadsheet. Demographic data, date of admission, diagnosis, procedures performed, HIV status, CD4 count in the last 3 months, outcomes, and date of discharge were abstracted.

**Definition:** a CD4 count of < 200 was considered immunodeficiency.

**Statistical analysis:** each data set was coded and analyzed using IBM SPSS-27 statistical software. HIV- and HIV+ patients as well as HIV+ patients with CD4 counts < 200 and > 200 were compared using outcome variables. The data were described using percentages and median [interquartile] ranges. Association between categorical variables was tested using Chi-squared, Mann-Whitney U test, and Fisher´s exact test as indicated. Continuous variables were compared with a 2-sample t-test. P-value < 0.05 was chosen to indicate statistical significance.

**Ethical consideration:** PMH (PMH 5/79(406-1-2017)) and Botswana's Ministry of Health and Wellness (HPDME 13/18/1 XI) Institutional Review Boards granted permission to conduct this research. Consent was waived for this retrospective medical record review. To ensure anonymity no patient identifier was collected.

## Results

### Characteristics of the study population

**Figure 1:**
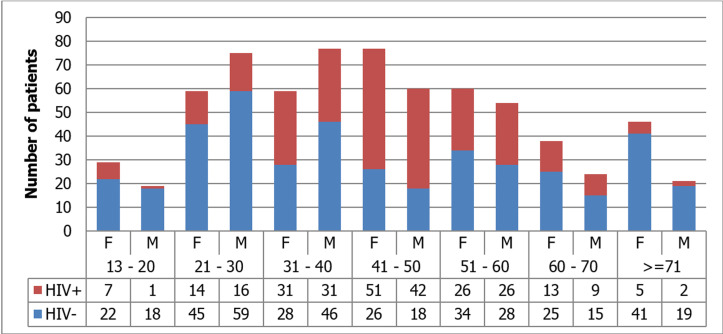
characteristics of the study population, PMH, August 2017 - July 2018

During the study period, a total of 698 HIV-status known patients were admitted with the diagnosis of surgical gastrointestinal diseases to the adult surgical wards. HIV+ patients contributed 274/698 (39.3%). Emergency admissions contributed 419(60.0%). Females were 365(53.6%). Age groups 13-20 years and > 41 years admissions were dominated by female patients, while age group 21-40 years was dominated by males. Males were admitted on emergency bases at a significantly higher rate than females, 69.1% vs. 51.9%, p=0.001. Females constituted 147/274 (53.6%) of the HIV+ group. There was no statistically significant difference in HIV infection rate among females and males, 39.9% vs. 38.5%, p=0.690 (Figure 1).

### Pattern and burden of surgical gastrointestinal disease admissions

The three most common surgical gastrointestinal disease admissions were hepatopancreatobiliary (25.4%), appendix (23.5%), and gastro- duodenal diseases (11.9%). The common hepatopancreatobiliary disease admissions include 63(35.6%) symptomatic gallbladder stone diseases and 42(23.7%) obstructive jaundice of unconfirmed causes. Among hepatopancreatobiliary pathologies, only symptomatic gallbladder stone diseases were significantly higher in HIV- than HIV+ patients, 11.3% vs. 5.5%, p=0.008. The common gastro-duodenal disease admissions include 50 (60.2%) upper gastrointestinal bleedings, 9 (10.8%) gastritis, and 9 (10.8%) gastric outlet obstructions. The common small bowel disease admissions include 53 (89.8%) small bowel obstruction and 2 (3.4%) enterocutaneous fistulae. The common colorectal disease admissions include 22 (38.6%) colon cancers, 15 (26.3%) rectal cancers, and 8 (14.0%) large intestinal obstructions. Appendicular disease admissions include 164 (100.0%) appendicitis. Anal and perianal disease (n=78) admissions include 27 (34.6%) perianal infections and fistulae and 14 (17.9%) anal cancers. Among the patients with perianal diseases, 55/78 (70.5%) were HIV+. Trauma involving the gastrointestinal tract includes 27(62.8%) stab injuries and 16(37.2%) blunt injuries. Among trauma patients 11/43 (25.6%) were HIV+ and among non-trauma patients 263/655 (40.2%) were HIV+ (Table 1). There was no statistically significant difference in the prevalence of hepatopancreatobiliary, gastro-duodenal, small bowel, colorectal, appendicular diseases, and trauma cases among HIV- patients and HIV+ patients. HIV+ patients were significantly more affected by anal cancers (5.1% vs. 0.0%, p=0.001), anal warts (4.4% vs. 0.0%, p=0.001), and perianal infections and perianal fistulae (6.2% vs. 2.4%, p=0.010) than HIV- patients.

**Table 1:** pattern and Burden of the admissions, PMH, August 2017-July 2018

Pattern	Diseases	Burden
Hepatopancreatobiliary diseases (n=177)	Symptomatic gallbladder stone	63 (35.6%)
	Obstructive jaundice of unknown cause	42 (23.7%)
	Pancreatic tumors	19 (10.7%)
	Hepatic, Gallbladder and bile duct tumors	20 (11.3%)
	Pancreatitis	15 (8.5%)
	Cholecystitis	13 (7.3%)
	Common bile duct stricture	2 (1.1%)
	Hepatic abscess	2 (1.1%)
	Hepatic cyst	1 (0.7%)
Gastro-duodenal diseases (n=83)	Upper gastrointestinal bleeding	50 (60.2%)
	Gastritis	9 (10.8%)
	Gastric outlet obstruction	9 (10.8%)
	Peptic ulcer perforation	7 (8.4%)
	Gastric cancer	3 (3.6%)
	Feeding gastric tube insertion	3 (3.6%)
	Foreign body in the stomach	2 (0.8%)
Small intestinal diseases (n=59)	Small bowel obstruction	53 (89.8%)
	Enterocutaneous fistula	2 (3.4%)
	Reversal of ileostomy	1 (1.7%)
	Prolapse of ileostomy	1 (1.7%)
	Small bowel tumor	1 (1.7%)
	Crohn’s disease	1 (1.7%)
Colorectal diseases (n=57)	Colon cancer	22 (38.6%)
	Rectal cancer	15 (26.3%)
	Large intestine obstruction	8 (14.0%)
	Lower intestinal bleeding	5 (8.8%)
	Reversal of colostomy	2 (3.5%)
	Prolapse of colostomy	2 (3.5%)
	Diverticulosis	1 (1.8%)
	Rectal prolapse	1 (1.8%)
	Constipation	1 (1.8%)
Appendix (n=164)	Appendicitis	164 (100.0%)
Anal and Perianal diseases (n=78)	Perianal infection & fistula	27 (34.6%)
	Anal cancer	14 (17.9%)
	Anal warts	12 (15.4%)
	Anal fissure	11 (14.1%)
	Hemorrhoids	10 (12.8%)
	Anal stenosis	2 (2.6%)
	Anal polyp	1 (1.3%)
	Anal incontinence	1 (1.3%)
Trauma (n=43)	Stab	27 (62.8%)
	Blunt	16 (37.2%)

Others (n=37): 19 abdominal masses– unspecified, 11 acute abdomen–unspecified, three diaphragmatic hernias, two splenic pathologies, and one each post Caesarian section intra-peritoneal retained swab, and tuberculosis peritonitis

### Outcomes of the surgical gastrointestinal disease admissions

A total of 63/698 (9.0%) major complications were recorded, including 43/698 (6.2%) deaths: 17/177 (9.6%) in hepatopancreatobiliary, 8/83 (9.6%) in gastro-duodenal, 5/59 (8.5%) in small intestine, 3/57 (5.3%) in colorectal, 1/164 (0.6%) in appendix, 3/78 (3.8%) in anal and perianal, and 6/37(16.2%) in “others” group admissions. Superficial SSIs and pneumonia contributed 15 (2.1%) and 2 (0.3%) respectively, while acute kidney injury, anastomotic leak, cholangitis, and wound dehiscence each contributed 1 (0.1%). The mortality rate was significantly higher in HIV+ than HIV- patients, 8.4% vs. 4.7%, p=0.048 (Table 2). The CD4 counts were available for 225 (82.1%) of HIV+ patients; 199/225 (88.4%) had a CD4 count of >200. Considering CD4 count < 200 as a cutoff point for immunodeficiency, there was no statistically-significant difference in the rates of overall complications,19.2% vs. 11.6%, p=0.338, superficial SSI, 0.0% vs. 5.3%, p=1.000, number of procedures, 46.2% vs. 56.8%, p=0.305, mortality, 19.2% vs. 8.5%, p=0.149, and median [IQR] hospital stay, 8 [2-11] vs. 7 [4-14], p=0.181 among HIV+ patients with CD4 counts < 200 and > 200 respectively.

**Table 2:** procedures, complications and hospital stay among HIV- and HIV+ patients, PMH, August 2017 - July 2018

Variables		HIV-	HIV+	p-value
Surgical procedures	Yes	217 (51.2%)	142 (51.8%)	0.868
	No	207 (48.8%)	132 (48.2%)	
Overall complications	Yes	34 (8.0%)	29 (10.6%)	0.248
	No	390 (92.0%)	245 (89.4%)	
Infectious complications	Yes	11 (2.6%)	6 (2.2%)	0.735
	No	413 (97.4%)	268 (97.8%)	
Mortality	Yes	20 (4.7%)	23 (8.4%)	**0.048**
	No	404 (95.3%)	251 (91.6%)	
Superficial SSI§(n=359)	Yes	9 (4.1%)	6 (4.2%)	0.971
	No	208 (95.9%)	136 (95.8%)	
Pneumonia	Yes	1 (0.2%)	0 (0.0%)	1.000
	No	423 (99.8%)	274 (100.0%)	
Acute kidney injury	Yes	1 (0.2%)	0 (0.0%)	1.000
	No	423 (99.8%)	274 (100.0%)	
Anastomotic leakα(n=240)	Yes	1 (0.6%)	0 (0.0%)	1.000
	No	158 (99.4%)	81 (100.0%)	
Cholangitis	Yes	1 (0.2%)	0 (0.0%)	1.000
	No	423 (99.8%)	274 (100.0%)	
Wound dehiscence§ (n=359)	Yes	1 (0.5%)	0 (0.0%)	1.000
	No	216 (99.5%)	142 (100.0%)	
Hospital stay: Median [IQR]		6[3-12]	6[3-12]	0.249

§ the denominator is the number of operated patients; α the denominator is the number of laparotomies with bowel suturing

## Discussion

This study investigated the pattern, burden and outcomes of surgical gastrointestinal diseases in a population with a high HIV infection rate. The prevalence of HIV infection among our admissions was high, 39.3%; females constitute 53.6% of the HIV+ group. Hepatopancreatobiliary, appendix, and gastro-duodenal diseases were the top three admissions respectively. Symptomatic gallbladder stones diseases were most common in HIV- patients. HIV+ patients had significantly higher rate of anal and perianal diseases. The mortality rate was higher in HIV+ patients.

The prevalence of HIV infection in surgical admissions was reported between 6.7% and 36.0% [2,21-25], and it is higher than the rate reported in the general population which ranges from 5.0% to 15.0% [2,22,26]. HIV infection prevalence of 39.3% in our admission reflects the higher prevalence of HIV infection (23.0%) [19] in our population and also it confirms the higher rate of HIV infection in our surgical gastrointestinal diseases admissions than the general population [27]. Our overall female-to-male admission ratio, 1.1: 1, is consistent with the reported 1: 1.4 to 1.4: 1 range [18,21]. HIV infection prevalence in female and male surgical admissions ranges from 21.0%-41.0% and 10%-36.0% respectively [2,18,21-24]. Botswana has a female HIV-infection prevalence of 20.8% and 15.6% in males [4]. Thirty-nine point nine percent (39.9%) HIV infection prevalence in our females falls in the reported range, and it relates to the higher HIV infection prevalence in our female general population. But a 38.5% prevalence of HIV infection in our male admissions was higher than in other studies; this could be partly due to the higher prevalence of HIV infection in our male general population. The age group and gender commonly affected by HIV infection vary depending on the HIV infection prevalence in the population and the sexual orientation of a population under study [2,11,18,21-25]. In our study age group 13–50-year females were more affected than males 59.8% vs. 39.0%; while age group >50 years males were more affected than females 57.6% vs. 30.6%. We found the highest HIV infection rates in the age group 41-50 years in both sex, 66.2% in females and 70.0% in males. This is consistent with the high prevalence of HIV infection in the same age group in our general population, 41.6% in females and 43.8% in males [27].

Surgical gastrointestinal diseases that include peptic ulcer disease, cholecystitis, pancreatitis, and appendicitis occur in the same frequency in HIV- and HIV+ patients [7,22,28]. We found a similar pattern of admissions for the same diseases, except for symptomatic gallstone disease which was significantly higher in HIV- patients and anal cancer, perianal infections and fistulae, and anal warts were significantly higher in HIV+ patients. HIV infection was reported more among non-trauma patients, 16.4%-34.0%, than among trauma patients, 12.7%-31.9% [3,28]. This is consistent with our findings, 40.2% for non-trauma and 25.6% for trauma admissions. The relatively higher percentages of both rates could be due to a high HIV infection prevalence in our population. Surgical patients presenting with infective processes in their pathology had the highest HIV prevalence [2,24,25]. Anal and perianal pathologies were reported as major surgical admissions in HIV+ patients [11,14]. Similarly, 70.5% of our admissions in anal and perianal pathologies were HIV+. Toxic mega-colon, bowel perforations secondary to Kaposi´s sarcoma or CMV, small and multiple liver abscesses, and biliary tree obstruction by CMV were reported in severely immunocompromised HIV+ patients [3]. This trend was not seen in our HIV+ patients, and this may be due to our “treat-all” guideline where all HIV+ citizen patients are eligible for free anti-retroviral therapy since 2016 [20]. Gonzalez *et al*. reported that HAART did not produce statistically significant differences in the rates of condylomata, fistula, hemorrhoid, and perianal abscess among HIV+ patients [29]. Our HIV- and HIV+ patients underwent a similar rate of surgical procedures, 51.2% and 51.8% respectively. This is consistent with the study by Martinson *et al*. [3] but it is in contrast to other reports that showed fewer surgical procedures in HIV+ patients [24] and more surgical procedures in HIV+ patients [18]. The rate of surgical procedures among our HIV+ patients with CD4 counts <200 and >200 did not differ; this was similarly reported by others [18]. The difference in the rate of surgical procedures among different studies is multifactorial that includes the type of the disease, antiretroviral treatment, CD4 count, viral load, availability of particular surgical service, and resources.

Many researchers reported the absence of significant differences in the rate of morbidity and mortality between HIV- and HIV+ patients [3,14,18]. The overall complication rates reported range 5.1%-14.3% in HIV- and 6.5%-40.0% in HIV+ patients [3,12,30]. In our study the overall complication rates fall in the reported ranges of 8.0% and 10.6% for HIV- and HIV+ patients respectively. Our overall SSI of 4.2% is lower than the 35.6% reported by Akoko *et al*. from Tanzania [21]. In HIV+ patients SSI was reported between 1.2%-21.4% [1,21]; our 4.2% finding for HIV+ patients falls at the lower end of the reported range. This may be partly due to the free HAART prescriptions for our HIV+ patients [20]. We found a significantly higher mortality rate in HIV+ than HIV- patients, 8.4% and 4.7% respectively, p=0.048; this is in contrast to other reports of the absence of significant difference [12,18,24,26]. Many studies show a significantly higher risk of SSI in HIV+ patients with a CD4 count <200 [1,15,17,30,31] but this was not the case in our study; this may be due to the small number of HIV+ patients with a CD4 count < 200 combined with a free HAART prescription and a high rate of compliance in our population. Though the mortality rate among HIV+ patients with CD4 count < 200 is higher than those with CD4 count >200, it did not reach statistical significance, 19.2% and 8.5%, p=0.149. This is in agreement with a report by Chichom-Mefire *et al*. from Cameroon [12] but in contrast to Albaran *et al*. from the United States who reported higher mortality in CD4 count <200 [31]. Similar to previous studies we did not find a significant difference in the length of hospital stay among HIV- and HIV+ patients [18,24,25]. The hospital stay among our HIV+ patients was not affected by their CD4 count, this is similarly reported by Cacala *et al*. from South Africa [18]; while Chichom-Mefire *et al*. reported longer hospital stays for patients with CD4 counts < 200 [12].

This study has some limitations. The CD4 counts for 49 (17.9%) HIV+ patients were not retrieved; this could modify some of the results. Absence of follow-up data and considering limited outcome determinants are the shortcoming of this study. A few postmortem results were retrieved to determine the exact causes of mortality and it was not included in this study. The large number of HIV+ patients from a single institution and the prospective data collection mode is the strength of this study.

## Conclusion

The patterns of surgical gastrointestinal diseases are similar among HIV- patients and HIV+ patients except for symptomatic gallbladder stones which were significantly higher in HIV- patients, and anal cancer, anal warts, and perianal infections and fistulae were significantly higher in HIV+ patients. A significantly higher mortality rate in our HIV+ patients despite the “treat all” policy since 2016 in Botswana [20] is worth further investigation to find out the factors behind this and improve the life of these patients.

### What is known about this topic

- *The prevalence of HIV infection in surgical practice is higher than the prevalence in the general population;*
- *Surgical gastrointestinal diseases such as peptic ulcer disease, cholecystitis, pancreatitis, ischemic bowel disease, appendicitis, and diverticulitis occur in the same frequency in HIV- patients and HIV+ patients;*
- *Advances in antiretroviral therapy reduce viral proliferation and significantly decrease the rate of septic and opportunistic complications in HIV+ patients and improve their survival*.

### What this study adds

- *Symptomatic gallbladder stone diseases were significantly high in HIV- than in HIV+ patients;*
- *Anal and perianal diseases were significantly high in HIV+ than in HIV-patients;*
- *HIV+ patients had a significantly higher mortality rate than HIV- patients*.
